# Harnessing crosstalk between extracellular vesicles and viruses for disease diagnostics and therapeutics

**DOI:** 10.20517/evcna.2024.30

**Published:** 2024-07-07

**Authors:** Xinxi Zhu, Xiuhui Lin, Liang Hu, Liangxing Wang, Qingfu Zhu

**Affiliations:** ^1^National Engineering Research Center of Ophthalmology and Optometry, Eye Hospital, Wenzhou Medical University, Wenzhou 325027, Zhejiang, China.; ^2^Key Laboratory of Heart and Lung, The First Affiliated Hospital of Wenzhou Medical University, Wenzhou 325000, Zhejiang, China.

**Keywords:** Extracellular vesicle, exosome, virus, viral infection, diagnostics, therapeutics

## Abstract

Extracellular vesicles (EVs) are increasingly acknowledged as important mediators of intercellular communication, closely related to the occurrence and development of a variety of diseases. Numerous studies have demonstrated that EVs play a multifaceted role in the infection process of viral diseases, elucidating their ability to both facilitate viral spread and inhibit infection progression. These versatile entities not only enhance infection rates and widen the scope of viral infection through the transmission of entire viruses or viral genomes, but also trigger antiviral responses and prompt cytokine secretion near the infection site, thereby fortifying the host's defense mechanisms and safeguarding neighboring cells against infection. This complicated crosstalk between EVs and viral infections prompts a deeper exploration into their roles in potential clinical applications. In this review, we aim to encapsulate the recent advances in understanding the intricate interplay between viruses and EVs, shedding light on the mechanisms underlying this vesicle-to-virion crosstalk. Furthermore, we underscore the significance of harnessing this knowledge for diagnostic and therapeutic functions in combating viral diseases.

## INTRODUCTION

Extracellular vesicles (EVs) are minute, membranous particles released by cells into the extracellular space. These vesicles are ubiquitous, being generated by virtually all cell types, and they can be found in a range of bodily fluids, including blood, urine, and saliva^[1]^. Their significance lies in their critical roles in facilitating intercellular communication by ferrying bioactive molecules such as proteins, lipids, and nucleic acids between cells^[2-4]^. EVs are classified into several subpopulations mostly based on their physical properties and cell origins. Among the diverse origins of EVs, small EVs (sEVs) with a size range of 30-200 nm, especially exosomes, stand out as a particularly intriguing subtype, typically measuring between 30 to 150 nm in size^[5]^. Researchers are captivated by EVs due to their profound involvement in fundamental physiological processes, including the modulation of immune responses, facilitation of tissue regeneration, and regulation of embryonic development. The study of EVs has burgeoned in recent years owing to their immense potential in diagnostic, therapeutic, and investigative applications across a spectrum of physiological and pathological contexts^[6-9]^.

Based on biogenesis ways, EVs are mainly classified into microvesicles (MVs), apoptotic bodies (ABs), and exosomes. MVs are formed by the outward budding of the plasma membrane, while exosomes are produced via the multivesicular bodies (MVBs) pathway and are released when MVBs fuse with the cell membrane. ABs are usually generated through a process termed apoptotic cell disassembly. The tetraspanins (such as CD9, CD63, and CD81) and endosomal sorting complex required for transport (ESCRT) proteins (TSG101, Alix), as well as membrane Proteins (Flotillin-1, Annexins), are often used for EV characterizations^[10,11]^. The cellular machinery involved in producing EVs and viruses shares numerous similar characteristics. EVs are endogenous vesicles carrying various bio-messages to mediate communications between cells and tissues, while viruses are exogenous and infectious entities that exclusively reproduce within the living cells of organisms. Typically, viruses are composed of genetic material, which can be either DNA or RNA, encapsulated within a protective protein coat called a capsid^[12]^. Additionally, certain viruses may possess an outer envelope composed of lipids. Viruses rely heavily on cellular machinery systems to carry out vital functions, including metabolism and replication, despite their own enzymes and genomes^[13]^. Traditionally, individual viral particles have been regarded as the primary infectious units capable of causing infection and proficient in both intra- and inter-organismal transmission. However, the revelation of EVs during viral infections has gradually undermined this longstanding hypothesis, triggering a reconsideration of the role of cell-derived EVs in viral infections. This discovery prompts a compelling need for further exploration into the intricate interplay between viruses and EVs, offering new insights into viral pathogenesis and potential therapeutic interventions^[14]^.

The exploration of the intricate relationship between virus infection and EVs has revealed fascinating insights. It has been discovered that EVs play a multifaceted role in the viral infection process. Notably, they can serve as facilitators, enhancing the spread and dissemination of viruses on a large scale^[15]^. Simultaneously, EVs exhibit a remarkable capacity to shield recipient cells from viral attacks, thereby conferring a degree of resistance against viral invasion^[16]^. Table 1 summarizes the dual effect of EV components in viral infection described in recently published literature. This dual nature underscores the complexity of the interplay between vesicles and viruses in the context of infection dynamics. Consequently, this review underscores the critical role of EVs in the realm of viral infectious diseases. It highlights their involvement in both pro- and antiviral activities across various stages of the infectious cycle, encompassing aspects such as disease diagnosis and treatment strategies. Understanding the intricate mechanisms by which EVs modulate viral infections holds significant promise for advancing our knowledge of infectious diseases and developing targeted therapeutic interventions.

**Table 1:** Effect of EV components on infection process

**Effect of EVs on viral infection process**	**Component**	**Source**	**Virus**	**Effective mode**	**Ref.**
Acceleration effect	Syntenin	Host	HCV	Promote the release of E2-containing EVs and resist neutralization	^[17]^
	let-7b, miR-206, miR-122	Host	HCV	Upregulate BAFF expression in macrophages via TLR7 activation	^[18]^
	miR-21	Host	HBV	Suppress host innate immune response	^[19]^
	VP2 YPX3L	Virus	HAV	Interact with ALIX and promote non-enveloped viruses’ transmission	^[20]^
	Structural protein pX	Virus	HAV	Interact with ALIX and promote non-enveloped viruses’ transmission	^[21]^
	ORF3	Virus	HEV	Interact with TSG101 and promote non-enveloped viruses transmission	^[22]^
	Nef	Virus	HIV	Affect cholesterol metabolism, cause inflammation and multiple comorbidities	^[23]^
	VP40	Virus	Ebola virus	Induce apoptosis in recipient T cells and monocytes	^[24]^
	miR-146a	Host	EV71	Suppress type I interferon response	^[25]^
	CAR receptor	Host	Coxsackievirus B3	Contribute to the attachment of CVB3 to exosomes	^[26]^
	PEDV N protein	Virus	Porcine epidemic diarrhea virus	Antagonize type I IFN production	^[27]^
Antiviral effect	miR-423-5p	Host	Rabies virus	Suppress cytokine signaling 3 on type I interferon signaling	^[28]^
	IFN-a	Host	HBV	Induce the transfer of antiviral molecules from LNPCs to hepatocytes	^[29,30]^
	Viral E protein	Virus	Zika virus	Bind ZIKV-neutralizing antibodies to prevent infection enhancement	^[31]^
	MHC	Host	Lymphocytic choriomeningitis virus	Recognize foreign peptides in the groove of MHC	^[32]^

### EVs secreted by virus-infected cells accelerate the viral infection process

**Figure 1:**
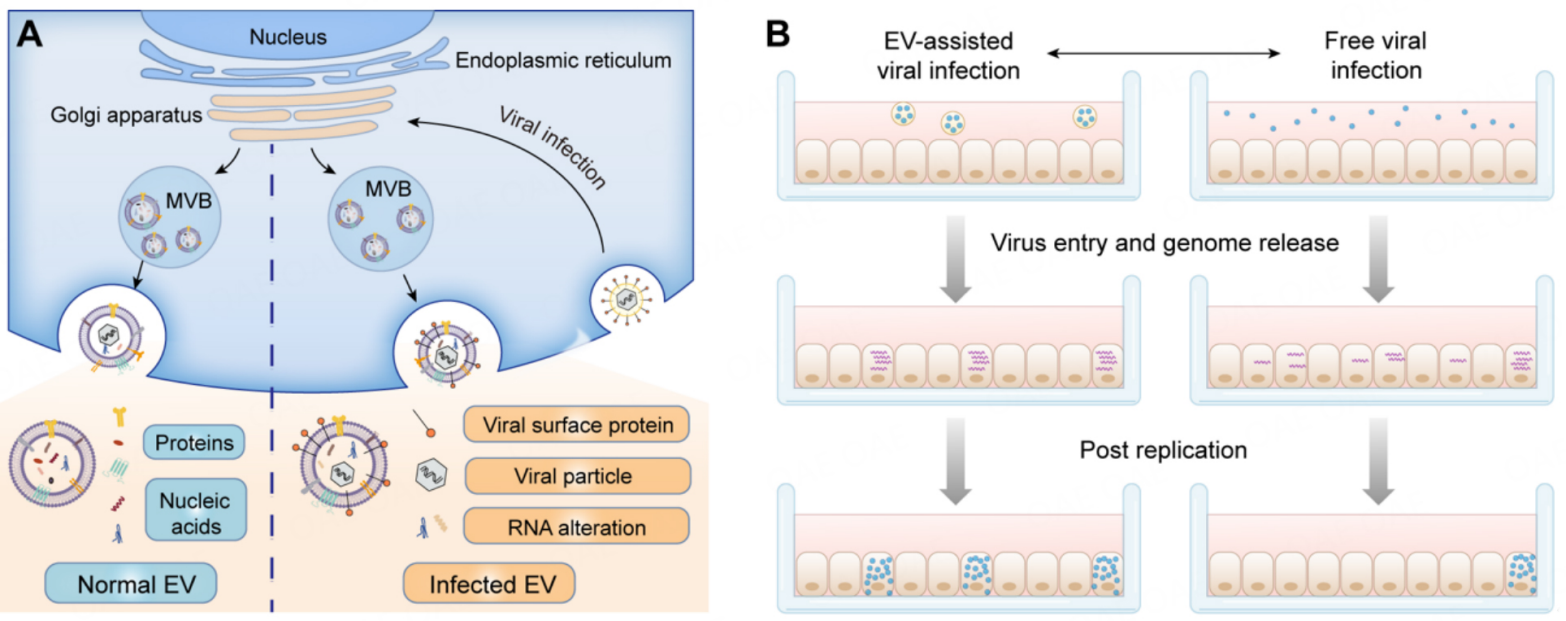
Interplay between virus and extracellular vesicles (EVs). (A) Interactions between virus and infected cell-derived EVs. (B) EV-encapsulated virus may enhance viral infectivity (adapted from ref^[14]^).

Recent research has unveiled a new concept: EVs may facilitate the transmission of viruses, both in vitro and in vivo, by integrating and transporting multiple intact viral particles or genomes into new recipient cells [Figure 1A]. This process, identified and termed vesicle-mediated en bloc transmission^[33]^, which challenges the conventional understanding of viral infection that typically involving the entry of individual viral particles into recipient cells. Increasing evidence suggests that viruses can be transmitted in vitro within viral clusters encapsulated within EVs. The infectious journey of these vesicles commences with their internalization into the endocytosis chamber of susceptible cells, followed by the transfer of multiple viral genomes into the host cell's cytoplasm^[34]^. This mechanism enables the simultaneous infiltration of numerous viral genomes, initiating infection within the same cell. This phenomenon often results in heightened infectivity compared to free virus particles, with a large number of infected cells secreting one or more viral particles, as observed through transfer electron microscopy (TEM)^[35]^. Furthermore, the efficient infectivity of EV-encapsulated viral particles is attributed not only to their low immunogenicity^[36]^ but also to their ability to evade antibody-mediated neutralization and prolong half-life in the extracellular space^[37-39]^. For instance, studies have demonstrated that HCV-infected cells secrete E2-coated exosomes, aiding viral escape from neutralization by both E2-specific antibodies and chronic-phase patient serum^[17]^. Additionally, pathogenic proteins carried by EVs can induce inflammatory responses. For example, Nef released from HIV-infected cells in exosomes may disrupt cholesterol metabolism in uninfected cells, contributing to systemic inflammation and the progression of HIV-associated comorbidities^[23]^. Moreover, following the delivery of exosome-decorated viruses, there is an increase in exosomal miR-21, miR-146a, and other immunosuppressive microRNAs, leading to the dysfunction of immune cells such as NK cells, T cells, and monocytes^[19,24,40]^, as well as a decrease in the expression level of antiviral cytokines including type I interferon^[25]^.

On the other hand, EV-encapsulated virus clusters may enhance viral infectivity, which can deliver multiple viral genomes simultaneously into the host cytosol [Figure 1B]^[14]^ . EV-encapsulated adeno-associated viruses (AAVs) serve as superior cardiac gene delivery vectors compared to free AAVs, demonstrating high therapeutic effects for neutralizing antibody resistance^[41]^. Moreover, the exosomal binding of virus particles not only enhances the infection rate but also enables the infection of cells lacking virus receptors^[35]^. This unique mode of infection may contribute to complications in non-recipient tissues after viral infection. For instance, tissues that do not express viral receptors may exhibit high viral loads during the acute infection phase. In a recent study, by binding to exosomes, Coxsackieviruses of Group B (CVB) can successfully infect receptor-negative host cells and establish productive infections^[26]^. The delivery of CVB virions carried by exosomes is dependent on the coxsackievirus-adenovirus receptor. Even macrophages can engulf the exosomal assembly of viruses and aid in the wide spread of viruses^[23]^. This particular mode of infection may contribute to complications in non-recipient tissues following viral infection^[18]^.

### How viruses are transmitted by EVs?

There is accumulating evidence suggesting that the production of infectious EVs may occur as a consequence of viral particles diverting the normal EV production pathway. Treatment with the exosome secretion inhibitors GW4869 and si-Rab27a to rabies virus-infected Vero cells reduces levels of extracellular and intracellular viral RNA^[42]^. Regarding the consensus on EV generation, it is widely recognized as a multifaceted process involving two invaginations of the plasma membrane and the subsequent formation of multivesicular bodies (MVBs) containing intraluminal vesicles (ILVs)^[4]^. This intricate process is intricately linked to plenty of cellular components, including RAS-related protein GTPase Rab, Syntenin-1, Tumor susceptibility gene 101 (TSG101), apoptosis-linked gene 2-interacting protein X (ALIX), syndecan-1, ESCRT (endosomal sorting complex required for transport) proteins, phospholipids, tetraspanins, ceramide, sphingomyelinase, and soluble N-ethylmaleimide-sensitive factor attachment protein receptor (SNARE) complex proteins^[43]^.

Initially, viral RNA is observed to co-localize with CD63, CD81, and CHMP4B proteins, which are known to be enriched in MVBs and play crucial roles in the biological processes of exosome formation^[44]^. Subsequent investigations revealed further detailed evidence. The biogenesis of coated hepatitis A virus (HAV) particles has been found to be associated with a variety of exosomal proteins, including the tetraspanin protein CD9 and dipeptidyl peptidase 4 (DPP4), as well as multiple endosomal sorting complex (ESCRT-III)-associated proteins necessary for transport^[45]^. Moreover, two tandem YPX3L late domains present in the HAV capsid protein VP2 were found to be associated with capsid stability and essential for viral release. These domains, along with the unprocessed structural protein VP1pX, have been shown to mediate interactions with ALIX protein^[20,21]^. In hepatitis E infection, the viral protein ORF3 has been demonstrated to interact with the ESCRT pathway to facilitate the budding and production of infectious vesicles^[22]^. Proteomic analysis of hepatitis C virus (HCV) particles identified multiple common markers of exosomes, such as syntenin, syndecan, and ALIX. It has been reported that syntenin, in conjunction with syndecan and Alix, functions as a key regulator controlling the formation of ILVs and the release of heat shock protein 70 (HSP70) as well as tetraspanin-containing exosomes from MVBs^[17]^. Experiments involving full-length release-defective HIV-1 showed that a loss of TSG101 recruitment or a mutation at the Alix interaction site would result in the failure of HIV-1 virus budding, thereby affecting transmission^[46]^. Collectively, this body of evidence strongly suggests that viruses exploit the exosome biogenesis pathway to exit infected cells.

In summary, both enveloped and non-enveloped viruses are previously thought to spread and infect a host as a single, independent viral particle. The discovery of EVs in viral transmission, as well as the discovery of “enveloped hepatitis A” and “enveloped enterovirus viruses”, has challenged this theory^[25,47]^. Moreover, it has been recognized that the non-enveloped viruses can acquire an envelope with the help of recipient cells during this process, and even gain a stronger infection ability than before^[45,48]^.

### EVs contribute to cellular resistance to virus infection

The impact of EVs secreted by infected cells on the course of infection is multifaceted. While the transfer of viral components via EVs may facilitate viral transmission, it is important to recognize that the host can leverage EV transport to induce an antiviral state within cells^[49]^. Notably, EVs serve as an effective means to transfer peptide antigens between cells and affect the subsequent specific immunity. For example, dendritic cells (DCs) cross-dressed with pMHC-I complexes from virus-infected cell-derived EVs can effectively activate CD8+ T cells and expand the immunologic process^[32,50]^. On the other hand, viral-modified EVs alert DCs with viral antigens to trigger adaptive immune responses^[44]^. The antiviral effect of EVs in the process of infection can relate to classical antiviral pathways, such as the interferon response. In the case of RABV infection, there is evidence that the upregulation of miR-423-5p in exosomes can inhibit RABV replication in MRC-5 cells. This inhibition is achieved by counteracting the suppressive effect of cytokine signaling suppressor 3 (SOCS3) on type I interferon (IFN) signaling. Furthermore, RABV infection has been shown to enhance the production of IFN-β, thereby exerting an inhibitory effect on RABV infection^[28]^. In addition, some cytokines and specific proteins are also involved in the process of fighting viral infection. IFN-a-stimulated human macrophages release exosomes containing antiviral mediators, such as the single-stranded DNA cytidine deaminase APOBEC3G (apolipoprotein B mRNA editing enzyme, catalytic polypeptide-like 3G), protecting human hepatocytes from HBV (hepatitis B virus) infection^[29]^. Similarly, in the context of Zika virus infection, EVs from infected cells have been observed to display viral E protein. This protein can bind ZIKV-neutralizing antibodies, effectively preventing infection enhancement^[31]^. Moreover, infection with Zika virus results in an increase in the expression of pro-inflammatory cytokines such as IL-1β, IL-6, and MCP1^[51]^. This heightened inflammatory response serves to combat the infection. In summary, while EVs secreted by infected cells can indeed aid in the spread of viral infection, they can also be harnessed by the host immune system to mount an antiviral defense. This dual role underscores the complexity of the interplay between viruses and host cells, highlighting the potential for therapeutic interventions aimed at modulating EV-mediated processes to combat viral infections.

### Advances in analytical tools for viruses and EVs

The progress of the research field of EVs and viruses is inevitably accompanied by the continuous advancement of corresponding analytical methods^[8,52-55]^. With the rapid development of the field, the separation and purification methods of nanoscale particles, as well as the follow-up detection and visualization tools, are constantly putting forward new requirements. Given the complex relationship between EVs and viruses, improved analytical methods are required for a deeper understanding of the interactions between viruses and EVs derived from infected cells. Currently, there are various techniques available for separating EVs, such as ultracentrifugation (UC), size exclusion chromatography (SEC), ultrafiltration (UF), flow field-flow fractionation, immunoaffinity capture, microchip-based methods, and the coupled methods^[56]^. UC is considered the gold standard approach for EV separation. However, the traditional UC method is inadequate for distinguishing between EV and virus particles due to their similar colloidal properties. In most cases, EV and virus were co-isolated by UC and then further purified by other purification methods. Among them, affinity purification is one of the most used purification methods due to the significant difference between EV membrane and nucleocapsid^[57]^. The antibody-based affinity-capture beads are applied to purify EVs and viruses. Most of these capture beads primarily target the tetraspanin proteins found on the surface of EVs (for example, CD63, CD81, and CD9), or specific viral proteins^[25,58,59]^.

**Figure 2:**
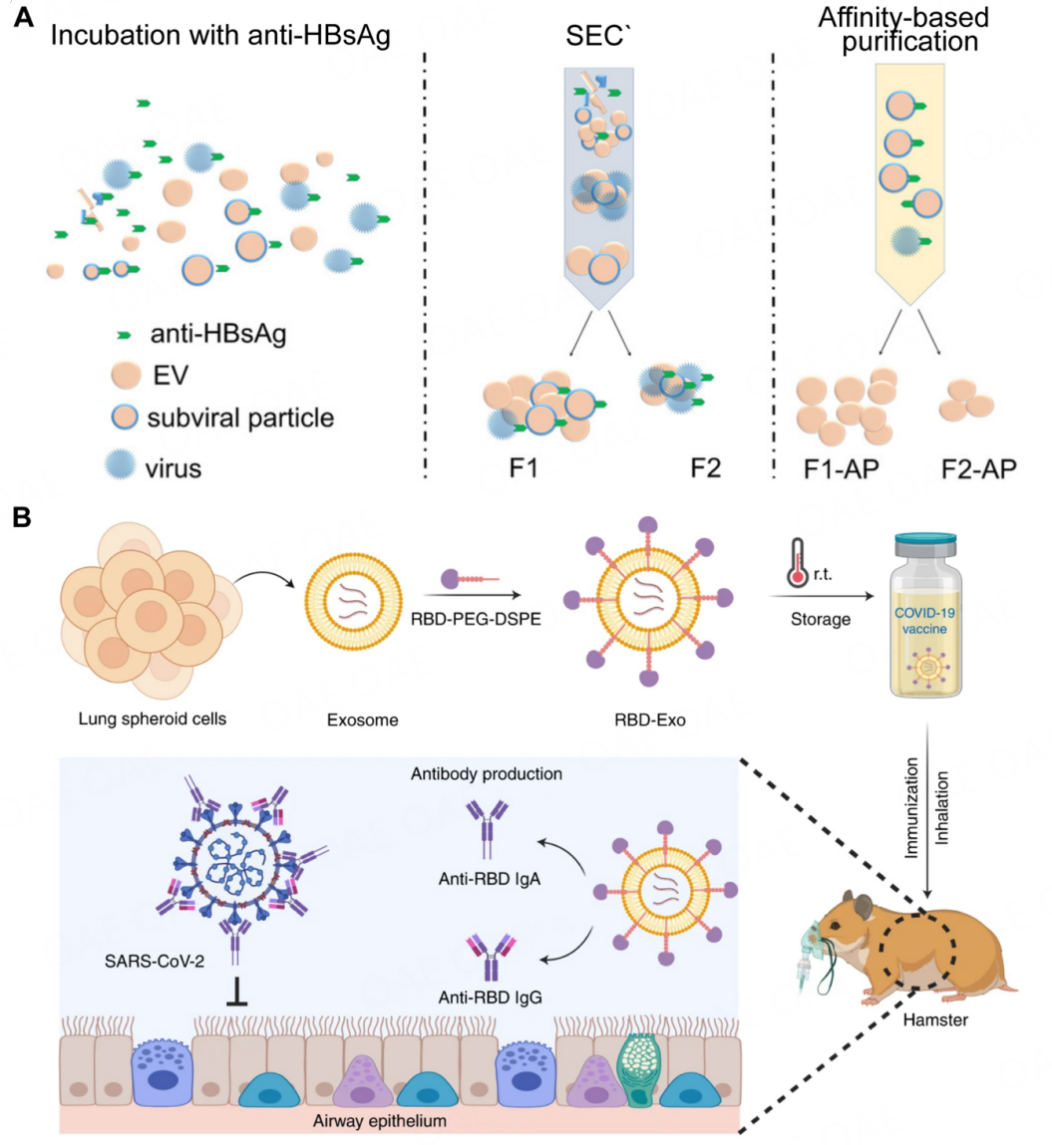
More advanced separation methods and future application directions between extracellular vesicles and viruses. (A) Schematic representation of virus removal from EVs through a combination method of SEC and 50% suspension of Protein A Agarose qEV columns (ref^[61]^). (B) Schematic representation of inhalation of the RBD-Exo VLP vaccine induces SARs-CoV-2 neutralization in hamsters and protects their lungs (ref^[78]^).

Moreover, density gradient centrifugation has also been used to isolate viruses and EVs. However, while gradients can be prepared to separate some more dense viruses from EV, co-contamination is expected for lower-density families of viruses. To have a better isolation, the ExoMAX/density gradient separation method is applied to isolate EVs from HIV virus. Briefly, cell supernatant is mixed with ExoMAX, a polyethylene glycol-based reagent, to precipitate particles, then the pellet is purified through iodixanol gradient and EVs are collected in 10.8 and 12.0 fractions^[60]^. Moreover, viral particles in EVs can be removed by viral antibodies. With hyperionized particles incubated with antibodies, the virus particles are removed using Protein A/G Magnetic Beads, followed by density gradient centrifugation to obtain EVs^[26]^. Similarly, 4G2 antibody can be used to isolate dengue virus and EVs. As an improved method, Protein A modified agarose-based size exclusion method combined with HBsAg capture is used to efficiently isolate hepatitis B from circulating EVs [Figure 2A]^[61]^. Additionally, the Exoview method enables the capture of multiple surface proteins of EVs and viruses, facilitating counting, particle size analysis, and even multi-protein colocalization analysis. This approach eliminates the need for separation and purification steps, allowing direct application in blood, urine, and cell culture medium with enhanced specificity, which stands as a prominent method for studying the complicated system composed of EVs and viral particles^[62]^.

### The role of infectious EVs in disease diagnostics and therapeutics

Numerous studies have demonstrated that EVs encapsulate specific disease markers both on and within their membranes, while alterations in miRNA profiles also hold diagnostic significance for disease occurrence and progression^[63-66]^. Furthermore, ongoing research is shedding light on the involvement of EVs in viral infectious diseases. Previous studies have shown that infected cells secret EVs carrying various viral miRNAs during viral infections^[67]^. In fact, the role of EVs extends beyond merely transporting viral genetic material, suggesting that altered EV components may serve as early indicators in disease diagnosis^[68,69]^. EVs expressing Epstein-Barr virus (EBV)-specific Latent Membrane Proteins (LMP), LMP1 and LMP2A, can be isolated from the medium of the NPC cell line C666-1. Additionally, in clinical settings, five cancer-related markers (LMP1, LMP2A, PD-L1, EGFR, and EpCAM) unequivocally differentiated patients from control groups when assessed via circulating EVs in blood circulation. Particularly, LMP1 and LMP2A, serving as latent membrane proteins specific to Epstein-Barr virus (EBV), offer a valuable means to identify EBV carriers and non-carriers^[70]^. In a retrospective study of COVID-19 patients, it has been found that the expression levels of tissue factors (TF) on EVs were positively correlated with mortality, and the presence of TF-positive EVs in the circulation may contribute to thrombosis with COVID-19 patients^[71]^. Furthermore, the expression of CD142, CD49e, CD69, and CD20 on circulating EV surfaces show differences between COVID-19 (-) pneumonia and COVID-19 (+) pneumonia, and CD142 also shows differences in mild and severe cases^[72]^. Altered miRNAs in exosomes can also be a biomarker of viral infection. For example, miR-122, let-7b, miR-206, and miR-155 levels have shown elevation in circulating EVs in patients with Hepatitis C Virus (HCV)^[18]^. Interestingly, let-7s is negatively correlated with the disease course during chronic HCV infection, which is considered suitable for assessing disease severity and as an indicator of chronic monitoring^[73]^.

In recent years, EVs have demonstrated remarkable efficacy in the treatment of viral infectious diseases^[74]^. Their superior characteristics have become apparent in two key areas: prevention and treatment. In prevention, EVs hold promise as components of vaccines. Significantly, the delivery of SARS-CoV-2 mRNA via EVs has demonstrated prolonged, dose-dependent induction of anti-spike and anti-nucleocapsid antibody responses, along with antigen-specific CD4+ and CD8+ T cell responses^[75]^. Moreover, EVs secreted by LPS-stimulated monocytic THP-1 cells can induce the increase in RANTES, IL-1β, TNF-, and IL-6 in spleen cells of healthy mice, and trigger a pro-inflammatory response. EVs secreted from THP-1, as adjuvants of HBsAg, can increase the concentration of IFN-, which will then enhance the cellular immune response of mice and accelerate the production of IgG antibodies^[76]^. Loading SARS-CoV-2 RBD protein (a key domain for viral attachment, fusion, and entry) onto EV membranes enables specific targeting of lung tissue and suppression of pseudoviral infection in vivo without the need for immune stimulation and triggers RBD-specific IgG and IgA as well as CD4+ and CD8+ T cells to neutralize SARS-CoV-2 [Figure 2B]^[77,78]^. The activated macrophage (M2)-derived EVs engineered with cardiac-targeting peptide (CTP) and platelet membrane (PM) show great potential for treating viral myocarditis toward clinical applications^[79]^.

Interestingly, even the EVs secreted by infected cells themselves contain the ability to inhibit the progression of the disease. Trans-activation response (TAR) element RNA in EVs from HIV-infected cells inhibits apoptosis by downregulating the expression of Bim and Cdk9 pro-apoptotic proteins^[80]^. Moreover, EVs isolated from serum during post-infection convalescence also have immune capabilities and could be a direction for vaccine development. Immunization of pigs with EV-enriched fractions from sera of PRRSV convalescence animals elicited specific humoral IgG immune responses and specific IFN- responses^[81]^. Some components of viruses also seem to help EVs function, and studies have shown that Arc, a homologous of retrovirus capsid protein (Gag), can increase the mRNA load of EVs, which, coupled with the low immunogenicity of EVs, may make EVs a good vehicle for drug delivery in vivo^[82]^. The gene editing ability of the prokaryotic CRISPR-Cas9 system has also shown its therapeutic role in viral infections^[83]^, but how to effectively deliver it to the body and better play the role warrants further consideration. Adenoviruses are the most commonly used vectors for CRISPR-Cas9 systems and are entering clinical trials^[84]^. At the same time, EVs also demonstrate the advantages of wrapping CRISPR-Cas9 systems and can target specific cells through surface modification^[85,86]^, and thus eliminating viral infection has become a new therapeutic direction^[87]^.

In addition, EVs from particular origins may show a strong ability to inhibit viral infection. It has been reported that exosomes secreted by mesenchymal stem cells (MSCs) can inhibit the RNA replication of hepatitis C virus^[88]^. Semen-derived exosomes may block HIV early protein transcriptional activator (Tat) recruitment and subsequent HIV-1 transcription, and promote transcriptional silencing of HIV-1 by disrupting NF-kB/Sp1/Tat circuitry^[89,90]^. Human placental trophoblast cells exhibit resistance to viral infection, and this resistance can be transferred through trophoblastic conditioned medium and trophoblastic exosomes^[91]^. This mechanism might represent a significant evolutionary adaptation to protect the developing fetus from viral threats. Additionally, macrophage-derived EVs may enter stem cells via TIM-1 and release IFN- to inhibit HBV activity^[30]^. Furthermore, exosomes of plant origin also display their unique role in viral resistance. Ginger-derived exosomal miRNAs, such as arlcv-miR-rL1-28-3p and aly-miR396a-5p, can potentially bind to multiple sites in the SARS-Cov-2 virus genome, thereby inhibiting viral gene expression and viral replication^[92]^. Plant-derived exosomes have a genome and proteome that are very different from human exosomes, and are easier to obtain and can be mass-produced, which could be a promising direction of reclamation. Moreover, studies have indicated that the existence of uninfected subpopulations of EVs secreted after viral infection can inhibit the viral infection process^[31,93]^. Thus, isolating these uninfected subpopulations is very important for therapeutic applications.

## CONCLUSION

Recent studies have demonstrated the multifaceted role of EVs in the viral infection process. These studies reveal that EVs possess the capability to both enhance and impede infection rates. Moreover, the dynamic interplay between viruses and EVs is evidenced by temporal fluctuations in exosomal compositions. Notably, viral infections prompt significant dysregulation of genes within exosome-related pathways. Furthermore, EVs sourced from specific origins and EV-based vaccines exhibit promising potential in fighting viral infection. Hence, investigations into the intricate relationship between EVs and viral infection processes hold promise for advancing disease diagnosis and treatment modalities.

However, current analytical techniques face several limitations, failing to completely separate EVs from viruses. Furthermore, there is no standardized method for producing large batches of EVs, highlighting the critical need for efficient separation techniques to advance the field. Additionally, establishing systematic qualification standards for EV products and obtaining regulatory agency approval are essential to ensure the safety of EV-based therapeutics. Despite the limitations, advancements in EVs-mediated drug systems in various clinical applications - particularly those harnessing viral infections to generate hybrid or engineered EVs - present a promising and viable future for therapeutic interventions.

## DECLARATIONS

### Copyright

© The Author(s) 2024.
